# Mapping Out the under-Recognized Burden of Human Infertility Linked to *Schistosoma haematobium* Infection

**DOI:** 10.4269/ajtmh.17-1016

**Published:** 2018-02-05

**Authors:** Charles H. King

**Affiliations:** Center for Global Health and Diseases, Case Western Reserve University School of Medicine, Cleveland, Ohio

In an article^1^ published in this month’s issue of the *American Journal of Tropical Medicine and Hygiene*, Patricia Woodall and Michael Kramer report on the ecological association between long-term exposure to *Schistosoma* infection and a woman’s odds of experiencing primary or secondary infertility in four East African countries—Ethiopia, Kenya, Tanzania, and Uganda. Links between schistosomiasis, (particularly urogenital schistosomiasis caused by *Schistosoma haematobium*) and either primary or secondary infertility have long been suspected based on past case reports^2,3^ and on limited cross-sectional community studies.^4–6^ However, the extent of the schistosomiasis-associated infertility problem has not been well quantified, and Woodall and Kramer’s new geographic landscape analysis defines the overlap between exposure (whether to *S. haematobium* or to *Schistosoma mansoni*) and the chances of experiencing infertility.

Infertility and all aspects of reproductive health are high priorities for every human culture. Problems with fertility are intimate and often not well captured in public health data because they are embarrassing or shameful to those who suffer from them. Because fertility problems are “not nice to talk about,” they are often understudied, undercounted, and not fully included in health policy analyses. Unfortunately, the influential Global Burden of Disease (GBD) project contends that such physical impairment is not the same as a disability and, therefore, GBD 2013 only credits infertility with an insignificant disability weight of 0.005–0.008. In translation, this means that, from the GBD’s disability adjusted life year perspective, infertility has very little impact on an individual’s disease burden, and that an infertile individual remains over 99% healthy.^7^

This is definitely not the case. At a minimum, one can infer from our societies’ large “willingness-to-pay” for assisted reproductive technology interventions (such as in vitro fertilization, which costs $12,000 or more per attempt) that infertility matters greatly to those who are affected and that they consider it a highly significant disability.^8^ There are substantial social costs as well—50% of couples’ infertility problems are male related, yet in practice, women are unfairly given the major share of blame when couples can’t conceive. The social, mental, and economic impact of this “gendered suffering” may prove to be devastating.^9^ Moderate to severe mental depression is common. In addition, negative societal attitudes towards infertile women can cause social stigmatization with consequent personal harm.^10^ Divorce or abandonment may ensue, and the woman’s social standing may be downgraded, including relegation to servant status and the loss of inheritance.^8,10^ In the absence of advanced medical testing, both partners in an infertile couple may turn to having sex with other partners to determine who is to blame for the infertility. This step involves increased risk of acquiring sexually transmitted infections (STI), including human immunodeficiency virus infection, for both partners. Some cultures or communities accuse infertile women of attempts to steal other women’s babies or of witchcraft. Such tagging can lead to fatal results.^11^

In sub-Saharan Africa there is a recognized “infertility belt” where subfertility rates of up to 30% are found (much higher than the worldwide average of 8–12%).^12–14^ Pelvic tuberculosis or STI such as *Chlamydia* and gonorrhea are frequently found in association with primary or secondary infertility.^13^ However, urogenital schistosomiasis has been mostly overlooked as a major contributing cause of infertility in at-risk populations. Symptoms of female genital schistosomiasis (FGS) and those of STI often overlap,^15^ and urogenital schistosomiasis patients frequently carry concurrent STI,^16^ which obscures the proximate contribution of FGS to their fertility problems. Given that its anatomic localization causes direct dysfunction in the reproductive tract, *S. haematobium* is the most likely “missing source” of infertility in sub-Saharan Africa. Schistosomiasis is also a chronic inflammatory condition of the whole body, further contributing to impaired fertility through abnormal hormonal or immune system regulation.

In their present analysis, Woodall and Kramer examine the impact of residence in areas endemic for *S. haematobium* as compared with residence in areas endemic for *S. mansoni*.^1^ In addition, they test the hypothesis that residence in a more highly endemic area is associated with greater odds of infertility than residence in a lower-transmission area. Their fertility data (focused on women 15–49 years old) were obtained from detailed, scientifically sampled demographic and health surveys performed in the targeted study countries. With the use of now standard statistical analyses for spatial associations and clustering, they identified the nonrandom distribution of infertility across the East African study landscape and the association of infertility with higher location-specific odds within *S. haematobium*–endemic zones. Across the study locations, residents in high-prevalence *S. haematobium* locations had significantly greater chances of experiencing infertility as compared with those living in high-prevalence *S. mansoni* locations, and as compared with residents of areas without much schistosomiasis. In stratified analyses, a duration of residence in a *S. haematobium* risk zone for > 10 years, or a history of exposure before age 10, were also significantly associated with greater odds of infertility.^1^

Now that we are more clearly aware of the problem, how do we undo or prevent the harm to reproductive health caused by *S. haematobium*? The World Health Organization and its strategic partners currently recommend implementation of national programs in all *Schistosoma*-endemic countries, focusing primarily on praziquantel treatment of school-age children,^17^ but with parallel recommendations to treat women of reproductive age in high risk populations.^18^ By the age of 10–12 years, school-age girls are known to already manifest symptoms of FGS.^19^ This makes their prompt treatment essential, because repeated school-age treatment is associated with early regression of many forms of *S. haematobium*–related pathology,^20^ with some continuing benefits into adult life.^5,21,22^ What is less certain is whether treatment of adolescents or young adults will be able to counter the effects of previously established FGS on their later fertility. Available evidence suggests that treatment of older age groups may be too little and too late to reverse the tissue damage already created by decades of past exposure to urogenital schistosomiasis.^23^

Without a doubt, FGS plays an important role in creating the infertility belt of sub-Saharan Africa. In this region, aggressive moves to obtain schistosomiasis control can now help to limit the lifetime uncertainty of childbearing success, permitting more confident birth interval planning with corresponding public health benefits in terms of maternal fitness, newborn health, and early childhood nutrition.^24^
